# Negative Magnetoresistance in Hopping Regime of Lightly Doped Thermoelectric SnSe

**DOI:** 10.3390/ma16072863

**Published:** 2023-04-04

**Authors:** Marija Zorić, Naveen Singh Dhami, Kristian Bader, Peter Gille, Ana Smontara, Petar Popčević

**Affiliations:** 1 Institute of Physics, Bijenička Cesta 46, 10000 Zagreb, Croatia; 2 Department of Earth and Environmental Sciences, Ludwig-Maximilians-Universität München, Theresienstraße 41, 80333 Munich, Germany

**Keywords:** hopping, metal-insulator transition, SnSe, negative magnetoresistance, positive magnetoresistance, Fermi liquid

## Abstract

Semiconducting SnSe, an analog of black phosphorus, recently attracted great scientific interest due to a disputed report of a large thermoelectric figure of merit, which has not been reproduced subsequently. Here we concentrate on the low-temperature ground state. To gain a better understanding of the system, we present magneto-transport properties in high-quality single crystals of as-grown, lightly doped SnSe down to liquid helium temperatures. We show that SnSe behaves as a p-type doped semiconductor in the vicinity of a metal-insulator transition. Electronic transport at the lowest temperatures is dominated by the hopping mechanism. Negative magnetoresistance at low fields is well described by antilocalization, while positive magnetoresistance at higher fields is consistent with the shrinkage of localized impurity wavefunctions. At higher temperatures, a dilute metallic regime is realized where elusive *T*^2^ and *B*^2^ resistivity dependence is observed, posing a challenge to theoretical comprehension of the underlying physical mechanism.

## 1. Introduction

Scientific interest in the SnSe compound is primarily driven by a recent report on its enormous thermoelectric potential (*ZT* = 2.6 at 923 K) [1]. This high *ZT* value has triggered a series of experimental [2,3,4,5,6] and theoretical studies [7,8,9,10,11,12]. However, the originally claimed figure of merit has not been reproduced [4,13]. Most studies have focused on the high-temperature properties of SnSe, well-documenting phase transition close to 800 K experimentally [1,3,14,15,16] and theoretically [7,17], as well as its pressure dependence [2,8,11,18]. It is established that crystalline SnSe is a semiconductor with a band gap close to 1 eV [13]. The transport properties reveal consistent p-type behavior [1,13,19,20,21]. Different doping degrees are reported to depend on the presence of oxidized Sn in the starting material for synthesis [22], resulting in Sn deficiency. Sn vacancies drive the Fermi energy towards the dispersive valence band, producing extra holes and intrinsically self-doping the crystal [23]. As a result, the Fermi level is positioned very close to the valence band.

Here we report magneto-transport properties of Bridgman-grown, lightly doped SnSe crystals with a carrier concentration of $2\times 10^{17}{\;\mathrm{cm}}^{-3}$. At the lowest temperatures below 10 K, the resistivity shows hopping behavior, followed by activated-like conduction in the intermediate temperature range. Above 70 K, the resistivity is metallic and follows elusive $T^{2}$ behavior, the microscopic understanding of which is still missing in diluted metals [24,25]. In the hopping regime, the magnetoresistance is negative at low fields, which is described by the magnetic field caused antilocalization mechanism. At higher fields, strong positive magnetoresistance due to the orbital shrinkage of impurity wave functions prevail.

There are two reports of magneto-transport on the semiconducting SnSe [20,26]. Both have higher concentrations of charge carriers, and, as a result, the hopping mechanism is not as pronounced as observed here. Only one study reports negative magnetoresistance at the lowest temperatures [26], attributed to the same mechanism as here. However, a different model was used there, which does not fit our data. A detailed study of the temperature dependence of magnetoresistivity and crystal anisotropy is also missing. Furthermore, we argue that the applicability of the standard Fermi liquid theory proposed in the literature [26] is questionable in this case due to the very small Fermi surface in the metallic regime.

## 2. Materials and Methods

SnSe was synthesized from Sn and Se granules, both 5N-grade. In order to remove inclusions of oxides, Sn was melted in an H_2_ atmosphere and slowly flown through a silica capillary into the carbon-coated fused silica ampoule prior to adding the untreated Se granules. A Se excess of 0.05 at.% was chosen instead of exact stoichiometry. The tapered ampoule of 13 mm inner diameter containing a total mass of 29.05 g was evacuated and fused once it reached a nominal pressure of 10^−6^ mbar. The ampoule was slowly heated in a vertical Bridgman furnace to a temperature slightly above the melting point of 873.7 °C [27] to allow the reaction. After 24 h rest period for homogenization, the ampoule was lowered through a temperature gradient of approx. 12 K/cm using a rate of 0.5 mm/h and finally cooled down to room temperature. The obtained ingot (see Supplementary Materials, Figure S1) was single-crystalline with the cleavage plane almost parallel to the growth direction (see Supplementary Materials, Figure S2). Cutting of the bar-shaped samples with dimensions of ∼2 × 2 × 8 mm^3^ with the long axis aligned along three perpendicular crystal directions was performed by a wire saw (WS 22, KD UNIPRESS, Warszawa, Poland) using a 50 µm thick tungsten wire and boron carbide powder (800 mesh) in a glycerol suspension to minimize surface damage of the samples. Adjusting the crystallographic orientation was performed using X-ray Laue backscattering technique and a special adapter to transfer the orientation to the wire saw. On as-cut surfaces, sharp reflection spots of the Laue images indicated the high structural quality of the single crystal as well as the gentle cutting process (see Supplementary Materials, Figure S3).

Since all three basic directions (*a*, *b*, and *c*) of an orthorhombic crystal exhibit the same 2D symmetry 2*mm* in Laue patterns, the absolute determination was obtained by comparing the experimental diffraction images with simulated ones using the LauePt program package [28]. There has been some confusion in the literature regarding the different space-group settings used for SnSe crystals. Sist et al. [29] compared the different settings and suggested that the low-temperature phase of SnSe should be described in space group *Pnma* (No. 62) with lattice parameters *a* > *c* > *b*. Accordingly, the cleavage plane should be indexed as (100). Refinement of the X-ray powder diffraction data from Mo-*K*_α1_ radiation using SnSe from the tip of the present crystal with the FullProf program package [30] yielded room temperature cell parameters of *a* = 11.498(1) Å, *b* = 4.152(1) Å, and *c* = 4.446(1) Å. For further details, please refer to the Supplementary Materials, Figure S4.

For electrical resistivity measurements, gold wires and Dupont 4929 N room-temperature silver paste were used to make electrical contacts to the sample on gold evaporated pads. Magnetoresistance measurements were carried out using the resistivity option of a Quantum design PPMS equipped with a 14 T superconducting magnet. The Seebeck coefficient was measured using a laboratory-made standard temperature-gradient technique [31]. Thermal conductivity was measured using the absolute steady-state heat flow method described in more detail in the literature [32]. The electrical resistivity under hydrostatic pressure was measured up to 2 GPa using a piston-cylinder pressure cell with Daphne 7373 oil as the pressure medium. The superconducting transition of the lead was used to estimate the pressure at low temperatures.

## 3. Results

### 3.1. Specific Heat

**Figure 1:**
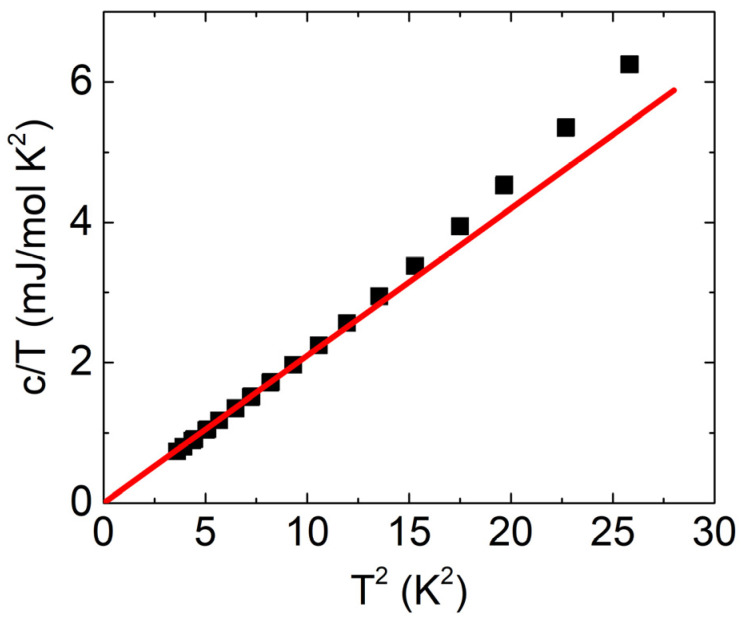
The low-temperature molar specific heat of SnSe in a c/T versus $T^{2}$ plot. The red line represents a fit to the data in the region between 2 K and 3 K. The values are calculated per mol of Sn_0.5_Se_0.5_ formula unit.

A convenient quantity to obtain an estimate of the electronic density of states (DOS) at the Fermi level $E_{F}$ and Debye temperature $\theta_{D}$ is the low-temperature specific heat *c*(*T*). In nonmagnetic materials, the specific heat is the sum of electronic and lattice contributions. At low temperatures (below ∼10 K), the lattice contribution can be approximated by the Debye model and can be expressed as $c_{latt}\left(T\right)=\beta T^{3}$, where the coefficient β is related to the Debye temperature $\theta_{D}^{3}=12\;\pi^{4}R/5\beta$, and R is the gas constant. The electronic contribution to the specific heat depends linearly on the temperature $c_{el}\left(T\right)=\gamma T$. The coefficient γ can be expressed as $\gamma =\left(\pi^{3}/3\right)k_{B}^{2}g\left(E_{F}\right)$, where $g\left(E_{F}\right)$ is DOS at $E_{F}$. The total specific heat at low temperatures can be written as $c\left(T\right)=\gamma T+\beta T^{3}$. To extract β and γ values, it is instructive to look at the specific heat in the form $c\left(T\right)/T=\gamma +\beta T^{2}$. Plotting $c/T\;\mathrm{vs}.T^{2}$ yields a straight line with intercept γ and slope β at low temperatures. The low-temperature molar-specific heat of SnSe is presented in Figure 1.

A linear fit of the data is possible below 3 K, as shown in Figure 1. At temperatures above this, the boson peak connected to diffusive damping becomes important, leading to deviations from the expected $\omega^{2}$ behavior of the vibrational density of states [33]. The Debye temperature extracted from the fit amounts to $\theta_{D}=(210\pm 2$) K, which agrees well with the previous reports in the literature [13,34]. The *γ* coefficient is zero within the error bar. This suggests that the density of states at the Fermi level is negligible, which is consistent with the absence of metallic behavior in the low-temperature resistivity.

### 3.2. Electronic Transport

**Figure 2:**
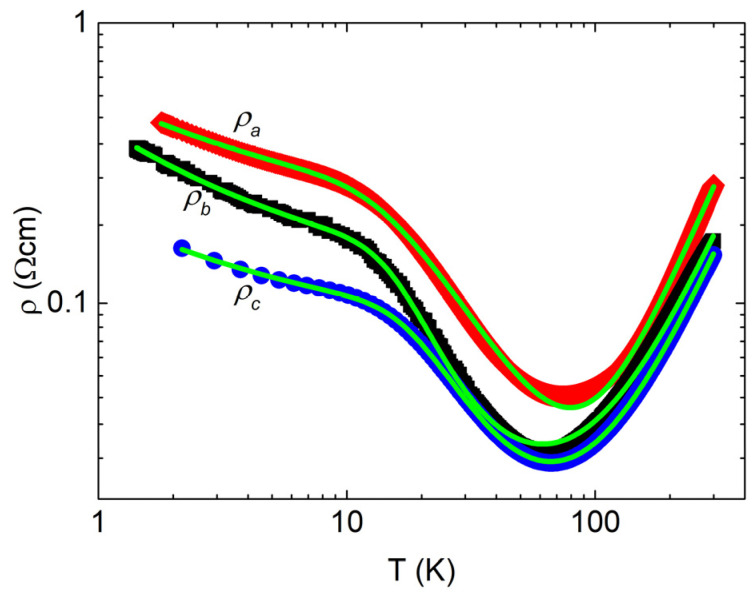
The temperature-dependent electrical resistivity of SnSe along three orthogonal crystallographic directions *a*, *b*, and *c*. Green lines represent fits to the model described by relation (4).

Figure 2 shows electrical resistivity along different crystallographic directions. As previously shown in the literature [1,13], there is an anisotropy between the stacking *a* direction and the *bc* plane, although it is somewhat smaller in this case. Starting from room temperature, the electrical resistivity exhibits metallic behavior, followed by a metal-to-insulator transition close to 70 K and an additional change in slope at 10 K. The value of electrical resistivity falls between 0.02 and 0.5 Ωcm, which is over three orders of magnitude above the typical values for metals. This indicates that the positive temperature coefficient at room temperature, consistent with vanishing specific heat coefficient γ, is the result of an exceedingly small Fermi surface. Above 20 K, the electrical resistivity in the *bc* plane is isotropic within the experimental error bar, while at lower temperatures, it is sampledependent even for the same crystal direction.

**Figure 3:**
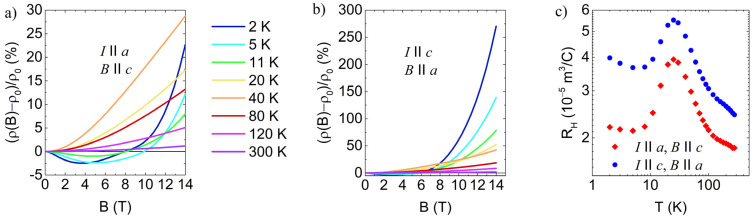
The magnetoresistance measured at different temperatures up to 14 T in case of (**a**) electric current along *c*-axis and magnetic field along *a*-axis, and (**b**) electric current along *a*-axis and magnetic field along *c*-axis. (**c**) The Hall coefficient measured in filed up to 2 T for electric current and field orientations presented in panels (**a**) and (**b**).

Figure 3 presents the magnetoresistance measured for the *a* and *c*-axis directions of electric current. For small magnetic fields, the magnetoresistance is negative for both directions up to 15 K, which corresponds to the temperature where the change in slope of the resistivity curve in Figure 2 is observed. At higher fields and temperatures, the magnetoresistance becomes positive. When the magnetic field is oriented along the stacking *a* direction, the magnetoresistance is an order of magnitude larger at low temperatures and high fields than for the field orientation in the *bc* plane. Negative magnetoresistance with a similar value has been reported in SnSe [19,26] in samples with a higher doping value that falls close to or into a degenerate semiconductor regime and is ascribed to weak antilocalization. However, such a large positive magnetoresistance, as observed in Figure 3b, has not been seen before.

Figure 3c shows the Hall coefficient measured for the same geometry as the magnetoresistance. The presented Hall coefficient corresponds to a charge carrier density of $1-3\times 10^{17}{\;\mathrm{cm}}^{-3}$, which is consistent with data reported in the literature [1,13]. The value is relatively constant compared to the variation observed at temperatures above the room temperature [1].

**Figure 4:**
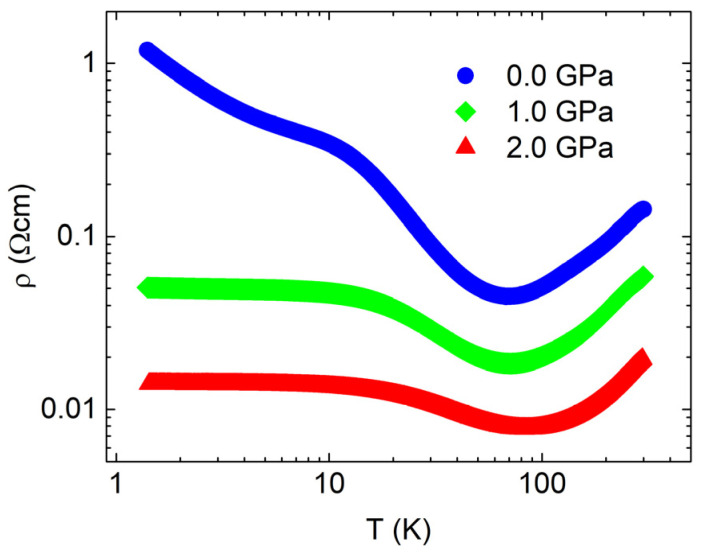
Electrical transport of SnSe crystal measured along *c* axis under hydrostatic pressure.

The electrical resistivity along the *c*-axis measured under hydrostatic pressure up to 2 GPa is shown in Figure 4. At a pressure of 2 GPa, the electrical resistivity is suppressed by order of magnitude at room temperature, while at low temperatures, the suppression is much larger, amounting to two orders of magnitude. A decrease in electrical resistivity, albeit to a somewhat lower degree, was also reported in metallic SnSe [21], where it was attributed to the emergence of a new Fermi surface under pressure. Metallization and superconductivity due to phase transitions under much higher pressures were also reported in semiconducting samples [8]. It should be noted that electrical resistivity presented in Figure 2 and Figure 4, along the same crystal direction, is measured on different crystal pieces.

## 4. Discussion

Three different regimes can be identified from the electrical resistivity curves in Figure 2. The first one spans a temperature range of up to 10 K, the second one from approximately 20 K to 50 K, and the third one above 100 K.

The Hall coefficient, which is inversely proportional to the charge carrier concentration, can be used to measure the doping level if we assume that it is solely caused by Sn impurities. The charge carrier density is found to have a range of $1-3\times 10^{17}{\;\mathrm{cm}}^{-3}$, which is comparable to or somewhat lower than [13,20,21] the value reported by other studies [1,13,19,20,21], indicating good quality (low amount of Sn vacancies) of our single crystals. To determine the degree of doping, we use the value of $Na^{3}$, where $a=\frac{\hbar^{2}\kappa }{{m\;e}^{2}}$ is the effective Bohr radius, and N is the impurity concentration. The semiempirical relation $Na^{3}≲0.02$ defines the doping range of lightly doped semiconductors where the impurity band is not metalized. The heavily doped region is usually defined as $Na^{3}\geq 1$, where the impurity band of electron donors (acceptors) merges with the conduction (valence) band. In the intermediate range of $0.02≲Na^{3}≲1$, the heavy doping criterion is still not satisfied, but the impurity states are not localized anymore, and the electrical resistivity shows metallic behavior down to the lowest temperatures. Considering that the charge carrier concentration obtained from the Hall coefficient is entirely caused by Sn impurities, using κ=15 [35] and $m=0.2*m_{e}$ [20,36], we arrive at $Na^{3}\approx 0.019$, which is on the brink of metallization. Thus, we conclude that our sample is in the light doping regime, very close to the metal-insulator transition (MIT). Consequently, we attribute the lowest temperature resistivity behavior to the hopping mechanism, followed by activated behavior in the intermediate temperature range and metallic behavior up to room temperature.

It is worth noting that Tayari et al. [20] demonstrate both metallic and hopping behaviors at low temperatures. Moreover, Wei et al. [13] report a higher concentration of charge carriers and a significant electronic contribution to the specific heat, which leads to the absence of a hopping region in low-temperature electrical resistivity. This is consistent with variations in the doping of different SnSe samples. Here we notice that upon applying hydrostatic pressure, electrical resistivity in the hopping regime becomes reduced, approaching metallization. The observed behavior is consistent with increased overlaps between localized states under pressure leading to metallization of the impurity band.

Variable range hopping is a widely used concept to explain low-temperature electrical resistivity in doped semiconductors [37,38,39,40,41], although its applicability may sometimes be limited to a relatively narrow temperature range [42,43,44,45,46]. It is well-established that in the case of variable range hopping, the electrical resistivity follows a particular behavior described by the equation:

(1) $$\rho \left(T\right)=\rho_{0}e^{{\left(T_{0}/T\right)}^{s}},$$

where $\rho_{0}$ is a temperature-independent prefactor, and s is 1/4 for Mott variable range hopping (VRH) in the 3D case and 1/2 for Shklovskii–Efros (SE) type hopping. The main difference between the two is the assumed density of states which is constant in Mott-type VRH and vanishes at the Fermi level due to Coulomb repulsion in the SE-type VRH [37].

**Figure 5:**
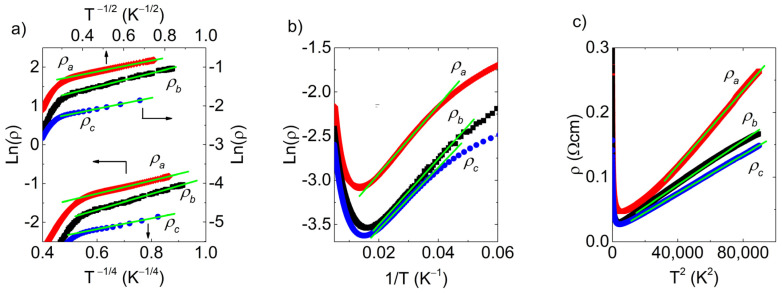
Electrical resistivity presented in the (**a**) $\mathrm{Ln}\left(\rho \right)\;\mathrm{vs}.T^{-\frac{1}{3}}$ plot, (**b**) $\mathrm{Ln}\left(\rho \right)\;\mathrm{vs}.T^{-1}$, and (**c**) $\rho \;\mathrm{vs}.T^{2}$. Green lines are guides for the eye.

As shown in Figure 5a, the temperature range of 1.5–10 K is too narrow to distinguish between Mott and SE-type VRH in this particular case. Furthermore, the proximity of the MIT, as discussed earlier, reduces the temperature variation in resistivity in the hopping regime, presenting an additional drawback. Therefore, to resolve the issue, measuring electrical resistivity down to the millikelvin range would be desirable. For the resistivity modeling, we progressed with s=1/4 since Mott VRH was argued to be a better description near MIT [44].

Consistently with the light doping regime, at intermediate temperatures between 30 K and 50 K, the electrical resistivity exhibits an activation-type behavior, as shown in Figure 5b. This behavior corresponds to the thermal activation of localized charge carriers, in this case, holes into the valence band. The activation behavior is described by the following relation:

(2) $$\rho \left(T\right)=\rho_{1}e^{E_{a}/k_{B}T},$$

where $\rho_{0}$ is temperature-independent prefactor and $E_{a}$ is characteristic activation energy. In this temperature range, the Hall coefficient shows a sharp decrease, indicating an increase in charge carriers. Above 100 K, the Hall coefficient has a more moderate temperature dependence, suggesting a relatively constant number of delocalized holes. This corresponds to metallic behavior in the electrical resistivity, which can be described by

(3) $$\rho \left(T\right)=\rho_{2}+A_{2}T^{2},$$

as demonstrated in Figure 5c. The $T^{2}$ behavior is usually connected to the electron–electron scattering within Fermi liquid theory. The specifics of the observed metallic regime are discussed below.

To describe the overall temperature dependence of the electrical resistivity, we use the following relation:

(4) $$\rho \left(T\right)={\left({\left(\rho_{0}e^{{\left(T_{0}/T\right)}^{1/4}}\right)}^{-1}+{\left(\rho_{1}e^{\frac{E_{a}}{k_{B}T}}\right)}^{-1}\right)}^{-1}+\rho_{2}+A_{2}T^{2}.$$

Relation (4) treats hopping and activation as parallel conduction channels. Although activated charge carriers cannot contribute to the hopping, the activation process does not prevent the hopping of still localized carriers, and hopping does not impair activation. However, metallicity can only be realized when a relatively constant number of charge carriers are activated to the band; thus, in relation (4), it occurs in series with the other conduction channels. By using relation (4), we obtain excellent qualitative agreement with the experimental results, as demonstrated in Figure 2.

It should be noted that relation (4) involves six fitting parameters. These parameters are presented in Table 1, along with the parameters obtained when each regime was analyzed separately, as shown in Figure 5.

Due to the relatively short temperature interval of each region, it is challenging to obtain accurate estimates for the parameters in relations (1)–(4). However, the variations in values presented in Table 1 give an indication of confidence in our results. The largest deviation occurs in the activation energy, which also happens to be the region of the graph in Figure 2, where the fit and experimental data show the poorest agreement. This discrepancy can be attributed to the weighting within the fitting procedure and the fact that the electrical resistivity has a minimum in the vicinity. The small activation energy suggests that the impurity band created by Sn vacancies is very close to the valence band, as suggested by [23]. The characteristic temperature of hopping, $T_{0}$, is approximately 2 K, which is unusually low and likely related to the proximity of MIT.

The electrical resistivity in the metallic and activated regimes shows small anisotropy between the out-of-plane *a* direction and the in-plane *b* and *c* directions. The in-plane anisotropy in this temperature regime is within the error bar. However, in the hopping regime, the observed anisotropy between in-plane and out-of-plane electrical resistivity curves is disrupted. While *a* and *c* directions preserve the original anisotropy, the electrical resistivity along the *b* direction is now closer to that along the *a* direction. This anisotropy disruption is likely related to the variation in the amount of Sn vacancies between different crystals and is more evident when comparing Figure 2 and Figure 5. Additionally, it was found that contact curing at elevated temperatures reduces resistivity and suppresses hopping and activated regimes, leaving only metallic behavior in the entire temperature range. This change can be described as the metallization of the impurity band, which eventually merges with the valence band. Additionally, this reveals annealing as a relatively simple doping mechanism for the SnSe system. At high doping, the Fermi level in SnSe enters the valence band, resulting in the formation of small Fermi surface hole pockets, consistent with both the experimentally measured and the theoretically calculated electronic band structure [9,13,20,36]. These pockets give rise to the observed metallicity.

### Magneto-Transport

There are several explanations for the negative magnetoresistance in the hopping regime. Fukuyama and Yoshida [47] proposed an approach applicable to Anderson localization near the metal-nonmetal transition. The mechanism is based on the repopulation of Zeeman split Anderson localized states and predicts a magnetic field dependence of $\frac{\Delta \rho }{\rho }\sim -T^{-2s}B^{2}$ in the low field limit. However, we find the approach taken by Raikh and Wessels [48] to be more appropriate here. They assume that direct tunneling between impurity levels and single scattering tunneling paths dominate the amplitude of a hop. In this case, the negative magnetoresistance is a result of the suppression of destructive interference. In amorphous semiconductors, multiple scatterings are more probable as stacking faults and grain boundaries serve as scatterers but do not provide localized states. Therefore, the approach taken by Raikh and Wessels is more suitable for crystalline semiconductors where impurities also create localized states, and only those impurities act predominantly as scatterers. This is the situation in single crystal SnSe, where Sn vacancies are dominant scatterers and also provide localized states. In the strong scattering limit and weak fields, this mechanism predicts a magnetic field dependence:

(5) $$\frac{\Delta \rho }{\rho }\sim -\frac{1}{T}B^{2}$$

for both Mott type and SE type hopping in the three-dimensional case.

**Figure 6:**
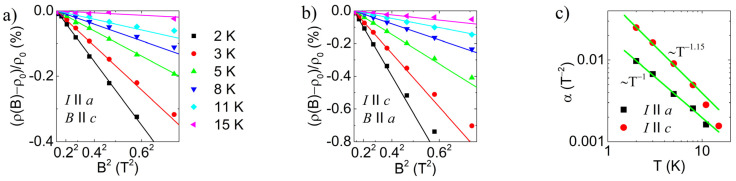
(**a**,**b**) Magnetoresistance in low field $B^{2}$ regime along different crystallographic directions. Lines represent fits to the experimental data up to 0.4 T. (**c**) Temperature scaling of magnetoresistance coefficient. Green lines are results of the fit.

Positive magnetoresistance, which is observed at higher magnetic fields, is commonly attributed to the effect of the orbital shrinkage of the impurity wave functions in magnetic fields [37]. Depending on the low or high field regime, or the type of hopping, different exponential magnetic field dependencies with appropriate temperature scaling laws have been predicted [37]. The combined negative and positive magnetoresistance reproduces the shape of the measured magnetoresistance curves qualitatively. However, we do not attempt combined fitting since Equation (5) is valid only in the low-field limit, and there is no simple universal relation that could describe magnetoresistance in the entire field range measured here. We found that magnetoresistance, presented in Figure 3, follows a $-\alpha B^{2}$ behavior in low fields. Good fits up to 0.4 T are obtained, as shown in Figure 6.

Figure 6c displays the temperature scaling of the $B^{2}$ coefficient for both crystallographic orientations. For the out-of-plane orientation of the electrical current (with the field perpendicular to it), we observe scaling with $T^{-1}$, consistent with the prediction by Raikh and Wessels [48]. In contrast, for the in-plane direction, we observe a slightly larger exponent of 1.15, which is, however, very close to the value of 7/6 predicted for the 2D case in the low-field, strong-scattering regime [48]. This confirms the electrical anisotropy of the system. We also note that both positive and negative magnetoresistance is stronger when the magnetic field is perpendicular to the conducting planes. This can be attributed to the localization length of impurity states being more extended in the plane and more strongly affected by the magnetic field. High electronic structure anisotropy was also confirmed through angle-resolved photoemission spectroscopy [36]. However, at temperatures above 10 K, scaling in Figure 6c is no longer obeyed since hopping is no longer the dominant mechanism, and the positive contribution becomes important even at low fields.

**Figure 7:**
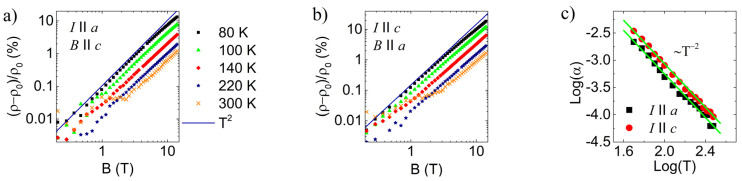
(**a**,**b**) Magnetoresistance in metallic regime along different crystallographic directions. Purple line represents $B^{2}$ dependence. (**c**) Temperature scaling of magnetoresistance coefficient. Green lines are results of the fit to $T^{-2}$ dependence.

At temperatures above the resistivity minimum, where $T^{2}$ behavior is observed, the magnetoresistance follows $B^{2}$ behavior, as shown in Figure 7. Slight deviation at the highest fields can be observed in the lower temperature range, indicating a crossover to saturation. The temperature scaling of the $B^{2}$ coefficient for both crystallographic orientations follow $T^{-2}$ behavior.

Recent research has attributed the quadratic temperature behavior observed in the metallic region of SnSe to the Fermi liquid-like electronic conduction, which is characterized by the dominant role played by electron–electron scattering [26].

The elusive $T^{2}$ behavior was recently reported in several systems, including KTaO_3_, Bi_2_O_2_Se, and SrTiO_3_ [24,25,49], which are characterized by vanishingly small Fermi surfaces where umklapp-like electron–electron scattering is supposedly not possible. Further theoretical work is needed to fully understand this phenomenon. Likewise, we cannot support a simple Fermi liquid scenario here due to the same reason, i.e., the small Fermi surface pockets. Additionally, Kohler’s rule, which is one important benchmark of a simple Fermi liquid, is not satisfied here. According to Kohler’s rule, the magneto resistivity coefficient should scale as $T^{-4}$ [50] which is not observed here, as demonstrated in Figure 7c. This was already indicated in the literature at the slightly higher doping value [20]. Instead, other scattering mechanisms, such as phonon-mediated electron–electron scattering [51], may be worth considering.

## 5. Conclusions

We conducted anisotropic magneto-transport measurements on high-quality single crystals of lightly doped semiconducting SnSe. At temperatures up to 10 K, the electrical resistivity follows hopping behavior due to impurities resulting from localized donor levels close to the top of the valence band caused by Sn vacancies. In this regime, we observe negative magnetoresistance at low fields, which we explain by magnetic field suppression of destructive interference. The temperature scaling of magnetoresistance coefficients is consistent with 3D-like transport along the stacking *a*-direction and 2D-like transport for in-plane direction. At higher magnetic fields, where the mechanism of shrinkage of localized wave functions is dominant, large positive magnetoresistance prevails. In the intermediate temperature range up to 50 K, the electrical conductivity is dominated by the thermal activation of localized holes to the valence band, with an activation energy equivalent to 40 K. At temperatures above 70 K, where all impurity levels are saturated, the electrical resistivity follows metallic $T^{2}$-like behavior with quadratic magnetoresistance. However, Kohler’s rule is not obeyed in this regime. This material thus belongs to the dilute metallic system class of materials, where elusive $T^{2}$ behavior exists in the limit of vanishingly small Fermi surfaces, presenting a challenge for the theoretical understanding of the scattering mechanism. The Fermi-liquid framework in its current form is arguably not applicable in this case.

## Figures and Tables

**Table 1:** Parameters of the fit for different regimes obtained from relations (1) to (3), while parameters obtained by relation (4) are given in italics for comparison.

Crystal Direction	$\rho_{0}\;\left(\Omega cm\right)$	$T_{0}\;\left(K\right)$	$\rho_{1}\;\left(\Omega cm\right)$	$E_{a}/k_{B}\;\left(K\right)$	$\rho_{2}\;\left(\Omega cm\right)$	$A_{2}\;\left(\mu \Omega cm/K^{2}\right)$
a	0.108	1.6	0.024	40.7	−0.007	3.02
a *	*0.163*	*1.3*	*0.078*	*45.3*	*−0.064*	*2.90*
b	0.054	2.1	0.015	36.4	0.022	1.68
b *	*0.047*	*2.2*	*0.0037*	*75.9*	*0.013*	*1.74*
c	0.048	1.4	0.015	34.4	0.015	1.49
c *	*0.043*	*1.5*	*0.0057*	*75.2*	*0.063*	*1.53*

* Data obtained using relation (4).

## Data Availability

Data presented here are available upon reasonable request to the corresponding author.

## Supplementary Materials

The following supporting information can be downloaded at: https://www.mdpi.com/article/10.3390/ma16072863/s1, Figure S1: As-grown SnSe crystal.; Figure S2: SnSe ingot cleaved along (100) plane; Figure S3: X-ray Laue back-scattering image of the (100) cleavage plane (white-beam Cu radiation; acceleration voltage of 45 kV at 33 mA; 50 mm crystal-film distance); Figure S4: X-ray powder diffraction pattern of phase-pure SnSe with 10 wt.% internal NIST Si640c standard(Mo-K_α1_ radiation). The agreement values are R_p_ = 10.7, R_wp_ = 10.2, R_exp_ = 5.71 and χ^2^ = 3.20.
