# Supplementary material for: Negative Magnetoresistance in Hopping Regime of Lightly Doped Thermoelectric SnSe
Source: Materials (Basel). 2023 Apr 4;16(7):2863. doi: 10.3390/ma16072863 (PMC10095749; doi:10.3390/ma16072863)
Supplement: Supplementary file 1 [file materials-16-02863-s001.zip › materials-2295953-supplementary.pdf]

Supplementary materials

to the article

Negative Magnetoresistance in Hopping Regime of Lightly Doped Thermoelectric SnSe

Marija Zorić, Naveen Singh Dhami, Kristian Bader, Peter Gille, Ana Smontara and Petar Popčević

The SnSe ingot in its as-grown state is displayed in Figure S1, with its conical tip being considered advantageous for selecting a single grain. The track of the cleavage plane (100) can be observed as a straight line almost parallel to the ingot's axis.

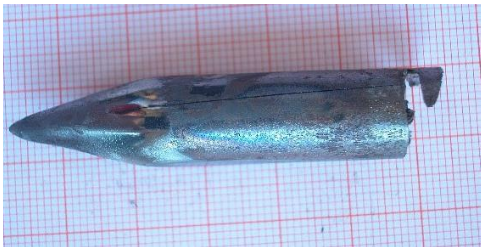

Figure S1. As-grown SnSe crystal.

After removing the tapered section of the ingot that froze first, the remaining single crystal could be cleaved easily along its entire length, as shown in Figure S2.

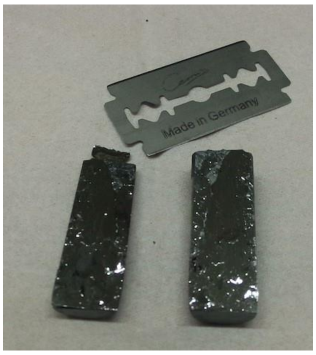

Figure S2. SnSe ingot cleaved along (100) plane.

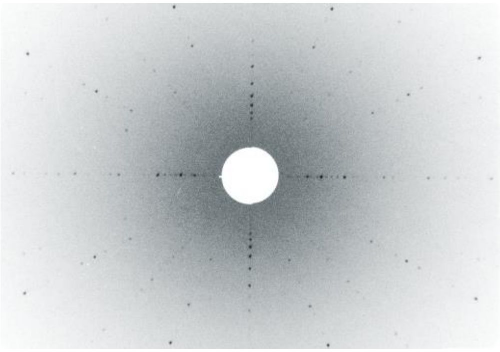

Figure S3. X-ray Laue back-scattering image of the (100) cleavage plane (white-beam Cu radiation; acceleration voltage of 45 kV at 33 mA; 50 mm crystal-film distance).

For X-ray powder diffraction, pieces of a phase-pure single crystal were cut, ground in an agate mortar in acetone and mixed with 10 wt.% NIST Si640c internal standard and finally filled in 0.3 mm silica capillaries. Three measurements were carried out on a STOE KRISTALLOFLEX diffractometer, using transmission mode in Debye-Scherrer geometry. The instrument was equipped with Mo-$K_{\alpha 1}$ radiation, a Ge(111) monochromator and a position-sensitive detector (PSD) with a resolution of 2θ = 0.1°/step. The measurement setup was 5° < 2θ < 60°, step width = 0.01°, 30 s exposure time per step, and acceleration voltage of 50 kV at 30 mA.

For Rietveld refinement, the program package FullProf [1] was used, with the pseudo-Voigt function. The powder patterns (see Figure S4) have a good fitting intensity and peak shape and no deviations of the published structure with the space group *Pnma* (No. 62) were found. The thermal vibration parameters were refined anisotropically. The lattice parameters were refined as a = 11.498(1) Å, b = 4.152(1) Å and c = 4.446(1) Å.

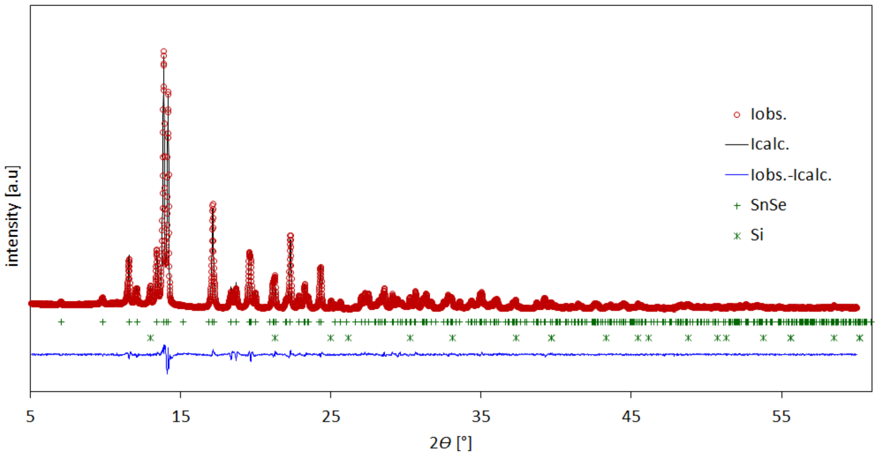

Figure S4. X-ray powder diffraction pattern of phase-pure SnSe with 10 wt.% internal NIST Si640c standard (Mo-$K_{\alpha 1}$ radiation). The agreement values are R_p = 10.7, R_{wp} = 10.2, R_{exp} = 5.71 and χ^2 = 3.20.

[1] Rodriguez-Carvajal, J. Recent Advances in Magnetic Structure Determination by Neutron Powder Diffraction. *Physica B* **1993**, *192*, 55-69.
